# Heterogeneous circulating miRNA profiles of PBMAH

**DOI:** 10.3389/fendo.2022.1073328

**Published:** 2022-12-13

**Authors:** Kazunari Hara, Masanori Murakami, Yoshihiro Niitsu, Akira Takeuchi, Masato Horino, Kumiko Shiba, Kazutaka Tsujimoto, Chikara Komiya, Kenji Ikeda, Mika Tsuiki, Akiyo Tanabe, Toshihiro Tanaka, Minato Yokoyama, Yasuhisa Fujii, Mitsuhide Naruse, Tetsuya Yamada

**Affiliations:** ^1^ Department of Molecular Endocrinology and Metabolism, Graduate School of Medical and Dental Sciences, Tokyo Medical and Dental University, Tokyo, Japan; ^2^ The Center for Personalized Medicine for Healthy Aging, Tokyo Medical and Dental University, Tokyo, Japan; ^3^ Department of Endocrinology and Metabolism, National Hospital Organization Kyoto Medical Center, Kyoto, Japan; ^4^ Department of Diabetes, Endocrinology and Metabolism, National Center for Global Health and Medicine, Tokyo, Japan; ^5^ Department of Human Genetics and Disease Diversity, Tokyo Medical and Dental University, Tokyo, Japan; ^6^ BioResource Research Center, Tokyo Medical and Dental University, Tokyo, Japan; ^7^ Department of Urology, Tokyo Medical and Dental University Graduate School, Tokyo, Japan; ^8^ Endocrine Center and Clinical Research Center, Ijinkai Takeda General Hospital, Kyoto, Japan; ^9^ Clinical Research Institute of Endocrinology and Metabolism, National Hospital Organization Kyoto Medical Center, Kyoto, Japan

**Keywords:** PBMAH, miRNA, Cushing syndrome, adrenal tumor, endocrinology

## Abstract

**Objective:**

Primary bilateral macronodular adrenal hyperplasia (PBMAH), a rare cause of Cushing syndrome, is often diagnosed as a bilateral adrenal incidentaloma with subclinical cortisol production. Circulating microRNAs (miRNAs) are a characteristic of adrenocortical adenomas, but miRNA expression in PBMAH has not been investigated. We aimed to evaluate the circulating miRNA expression in patients with PBMAH and compare them with those in patients with non-functioning adrenocortical adenoma (NFA) and cortisol-producing adrenocortical adenoma (CPA).

**Methods:**

miRNA profiling of plasma samples from four, five, and five patients with NFA, CPA, and PBMAH, respectively, was performed. Selected miRNA expressions were validated using quantitative RT-PCR.

**Results:**

PBMAH samples showed distinct miRNA expression signatures on hierarchical clustering while NFA and CPA samples were separately clustered. PBMAH was distinguished from the adenoma group of NFA and CPA by 135 differentially expressed miRNAs. Hsa-miR-1180-3p, hsa-miR-4732-5p, and hsa-let-7b-5p were differentially expressed between PBMAH and adenoma (*P* = 0.019, 0.006, and 0.003, respectively). Furthermore, PBMAH could be classified into two subtypes based on miRNA profiling: subtype 1 with a similar profile to those of adenoma and subtype 2 with a distinct profile. Hsa-miR-631, hsa-miR-513b-5p, hsa-miR-6805-5p, and hsa-miR-548av-5p/548k were differentially expressed between PBMAH subtype 2 and adenoma (*P* = 0.027, 0.027, 0.027, and 1.53E-04, respectively), but not between PBMAH, as a whole, and adenoma.

**Conclusion:**

Circulating miRNA signature was identified specific for PBMAH. The existence of subtype-based miRNA profiles may be associated with the pathophysiological heterogeneity of PBMAH.

## Introduction

Primary bilateral macronodular adrenocortical hyperplasia (PBMAH) is a rare cause of ACTH-independent Cushing syndrome. It is usually identified by adrenal imaging and is characterized by bilateral enlarged adrenal masses (symmetric or asymmetric) composed of multiple bilateral macronodules (>10 mm) with hyperplasia and/or inter-nodular atrophy (1). PBMAH is being diagnosed at an increasing rate due to the incidental detection of clinically mild or asymptomatic cases during abdominal imaging performed for unrelated reasons. Currently, PBMAH is characterized by high clinical heterogeneity, with respect to both severity of cortisol excess and morphological appearance of the adrenals (2). A large cohort study reported that among the patients with PBMAH, 43% had clinically overt Cushing syndrome and 47% had a subclinical phenotype (3). With the development of gene sequencing technology, gene mutations related to familial and sporadic PBMAH, such as *ARMC5* mutations have been discovered (3–7). However, the involvement of *ARMC5* inactivation during PBMAH pathogenesis is not fully understood (8). Patients with PBMAH can generally be grouped into two categories: those with multiple adenomas and inter-nodular atrophic cortical tissue, and those with diffuse hyperplasia and no residual normal or surrounding atrophic adrenal cortex (9, 10). Although it was reported that some patients with familial PBMAH harboring *MEN1* and *APC* mutations showed the phenotype of the first group (9), the detailed relationship between the sub-groups and their clinical and molecular features remains to be elucidated.

MicroRNAs (miRNAs) are small, non-protein-coding RNA molecules that are 16–24 nucleotides long in their single-stranded mature form. More than 50% of human protein-coding genes are predicted to be modulated by miRNAs (11). miRNAs, as epigenetic regulators, are mainly involved in the post-transcriptional regulation of gene expression (11). The development of next-generation sequencing techniques has provided rich information on miRNA profiles in adrenocortical tumor tissues. For example, miRNAs that play functional roles in adrenal tumorigenesis, including benign and malignant adrenocortical tumors have been identified (12). Further, miRNA profiles distinct from that of tumor tissues were observed in benign and hyperfunctioning adrenocortical tumors such as aldosterone-producing adenoma (APA) (13) and cortisol-producing adenoma (CPA) (14). In addition to the evaluation of miRNA expression in tumor tissues, recent studies have detected specific circulating miRNAs in blood samples of patients with APA (15) and CPA (16). Thus, circulating miRNA signatures of hyperfunctioning adrenocortical tumors have been suggested to be useful as non-invasive biomarkers. They may provide insights into the pathophysiological roles of circulating miRNAs in hyperfunctioning phenotypes. miRNA expression of bilateral adrenal hyperplasia has been examined (17, 18). Recently, specific miRNA profiles of sporadic and familial PBMAH tissue samples have been reported and their implications in pathogenesis have been suggested (19). However, circulating miRNAs of patients with PBMAH have never been reported. The aim of this study was to elucidate the signatures of circulating miRNAs specific in patients with PBMAH compared to those in patients with other forms of adrenocortical tumors, including CPA and non-functioning adenoma (NFA). miRNAs that are distinct in PBMAH samples can distinguish them from other forms of adrenocortical tumors and would provide new insights into the unique pathophysiological features of PBMAH.

## Materials and methods

### Patient cohort

The study protocol was approved by the Ethics Committee for Human Research at Tokyo Medical and Dental University (Tokyo, Japan) (G2020-027), Kyoto University (Kyoto, Japan) (G1186-3), and the NHO Kyoto Medical Center (19-005, 18-008). Written informed consent was obtained from all the participants. Plasma samples were obtained from four patients with NFA, five patients with CPA, and five patients with PBMAH. The hormonal profile was examined in all cases, and the diagnosis of hypercortisolism was based on the current guidelines (20, 21). We included only one patient with overt Cushing syndrome in the CPA and PBMAH groups. The clinical characteristics of the three cohorts are summarized in **Table 1**. Patients with PBMAH had no family history of endocrine disease including PBMAH and suggested sporadic forms. Plasma samples were collected prior to surgery in operated cases.

**Table 1:** Patient characteristics.

Sample number	Sex	Age at blood sampling	BMI (kg/m2)	Type	Hormonal activity	Basal ACTH (pg/ml)	Basal Cortisol (μg/dl)	Cortisol after LDDST (μg/dl)	UrinaryFree Cortisol (μg/day)	DHEAS(ng/ml)	Tumor diameter (mm)
1	M	60	22.2	NFA	–	19.5	14.5	1.3	14.2	392	12
2	F	64	27.1	NFA	–	8.3	8.4	1.2	38.3	140	27
3	F	66	19.6	NFA	–	22.1	20.1	1.2	32.5	1532	35
4	F	62	21.8	NFA	–	6.4	7.5	0.9	19.8	302	18
5	F	57	20.6	CPA	SCS	2.6	6.3	5.2	21.2	<50	20
6	F	53	21.5	CPA	SCS	3.2	11.3	11.9	15.4	160	24
7	F	50	23.6	CPA	CS	1.0	18.3	19.8	118	<50	27
8	M	72	22.4	CPA	SCS	11.2	10.2	5.7	33	213	43
9	F	53	27.3	CPA	SCS	6.3	12.1	6.8	16.5	<50	30
10	M	67	22.3	PBMAH	SCS	2.00	18.7	15.5	151	188	R: 39, L: 54
11	F	73	28.9	PBMAH	CS	1.00	19.5	24.4	155	<50	R: 42, L: 31
12	F	77	18.2	PBMAH	SCS	1.9	11.3	12.6	75.6	<50	R: 18, L: 42
13	M	72	26.9	PBMAH	SCS	5.2	10.1	8.2	54.6	115	R: 58, L: 49
14	M	44	22.8	PBMAH	SCS	4.8	6.08	3.15	176	52	R: 39, L: 19

M, male; F, female; BMI, body mass index; NFA, non-functioning adenoma; CPA, cortisol-producing adenoma; PBMAH, primary bilateral macronodular adrenal hyperplasia; SCS, subclinical Cushing syndrome; CS, Cushing syndrome; LDDST, low-dose dexamethasone suppression test; DHEAS, dehydroepiandrosterone sulfate.

### Sample preparation

EDTA-anticoagulated blood was collected from the patients in the morning and processed for plasma isolation immediately after blood collection. Plasma was obtained by centrifuging the whole blood at 1000 × *g* for 10 min at 4°C. All extracted plasma samples were stored at −80°C until further application. Total RNA was isolated from plasma using the miRNeasy Micro Kit (QIAGEN, Valencia, CA, USA).

### miRNA profiling analysis

The total RNA obtained from each sample was subjected to a sequencing library construction procedure using the QIAseq miRNA Library Kit (QIAGEN, Valencia, CA, USA) according to the manufacturer’s protocols. The QIAseq miRNA Library Kit integrates a unique molecular index (UMI) system, which enables accurate counting of unique miRNA molecules in samples. The quality of the libraries was assessed using an Agilent 2100 Bioanalyzer High Sensitivity DNA Kit (Agilent Technologies, Santa Clara, CA, USA). The equally pooled libraries were sequenced using NextSeq 500 (Illumina, Inc., San Diego, CA, USA) as 76-base-pair (bp) single-end reads.

The QIAseq miRNA library kit adopts a UMI-system, enabling unbiased and accurate quantification of mature miRNAs. The original FASTQ files generated by NextSeq were uploaded to the Qiagen GeneGlobe Data Analysis Center (https://geneglobe.qiagen.com) and aligned to miRBase v21 (http://www.mirbase.org) and piRNABank (http://pirnabank.ibab.ac.in/). All reads assigned to a particular miRNA or piRNA were counted and the associated UMIs were aggregated to count unique molecules. A matrix of the UMI counts of miRNA or piRNA was subjected to downstream analyses using the StrandNGS 3.4 software (Agilent Technologies, Santa Clara, CA, USA). UMI counts were quantified using the trimmed mean of M-value (TMM) method (22). miRNAs were annotated using miRBase (Release 21).

### miRNA qRT-PCR

Reverse transcription (RT) was performed on the miRNAs using a TaqMan Advanced miRNA cDNA Synthesis Kit (Applied Biosystems, Thermo Fisher Scientific, Waltham, MA, USA) according to the manufacturer’s protocol. The protocol consisted of four steps: (1) addition of a poly(A) tail, (2) adaptor ligation, (3) reverse transcription to cDNA, and (4) miRNA amplification. The converted cDNA templates were diluted for miRNA expression analysis using the QuantStudio 7 Flex Real-Time PCR System (Applied Biosystems, Thermo Fisher Scientific). The Ct values were converted into copy numbers (copy no. = 2^(−Ct)) and normalized to an endogenous reference gene. The candidate endogenous reference miRNAs were selected from the TaqMan Advanced miRNA Assays white paper (Applied Biosystems, Thermo Fisher Scientific) and evaluated for stable expression across miRNA-Seq data using the following criteria: (a) high read count in all samples and (b) no inter-group differential expression (*P*-value < 0.05). In addition, Normfinder algorithms (23) determined hsa-miR-16-5p to be the most stable endogenous normalizer. Thus, we selected it as an internal reference as previous articles used (24–27).

### miRNA pathway analysis

Functional pathway analysis for differentially expressed miRNAs was conducted by miR+Pathway, a pathway database that integrates and provides visualization of the 8882 experimentally validated human miRNA-target interactions and 150 KEGG pathways (http://www.insect-genome.com/miR-pathway) (28).

### Statistical analysis

The set of differentially expressed miRNAs were determined by miRNA-profiling analysis. The threshold of the absolute value for fold change was set as ≥ 2 and that of the adjusted *P*-value, estimated using a moderated *t*-test followed by Benjamini–Hochberg multiple testing corrections, was set at < 0.05. For the identification of differentially expressed miRNAs by qRT-PCR tests between two groups, Student’s *t*-test and Mann–Whitney *U* test were performed based on the results of the Shapiro–Wilk normality test. For the identification of those between the three groups, one-way ANOVA followed by Tukey’s post Hoc test or Kruskal–Wallis test followed by Dunn test were used based on the results of Shapiro–Wilk normality test. A *P*-value < 0.05 was considered statistically significant. The diagnostic applicability of miRNAs in adenoma (NFA and CPA) and PBMAH was analyzed by receiver operating characteristic (ROC) curves.

## Results

### Demographics

The clinical characteristics of 14 patients (five men and nine women with an average age of 62 ± 10 years and an average body mass index (BMI) of 23.2 ± 3.2 kg/m^2^ during blood collection) with adrenal diseases are summarized in **Table 1**. Our cohort included four patients with NFA (one man and three women with an average age of 63 ± 2 years and an average body mass index (BMI) of 22.7 ± 3.2 kg/m^2^ during blood collection), five with CPA (one man and four women with an average age of 57 ± 9 years and an average body mass index (BMI) of 23.1 ± 2.6 kg/m^2^ during blood collection), and five with PBMAH (three men and two women with an average age of 67 ± 13 years and an average body mass index (BMI) of 23.8 ± 4.2 kg/m^2^ during blood collection). One patient was diagnosed with overt Cushing syndrome and four patients with subclinical Cushing syndrome in both the groups of patients with CPA and PBMAH each.

### miRNA profiles and phenotype correlation

**Figure 1:**
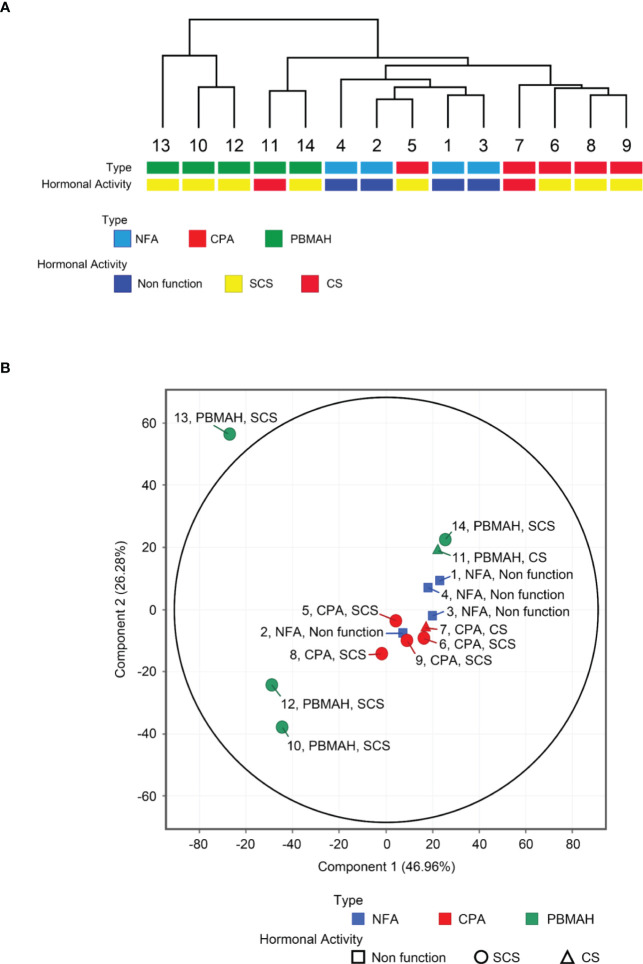
Circulating miRNA expression signatures of NFA, CPA and PBMAH. Unsupervised hierarchical clustering analysis **(A)** and principal component analysis **(B)** of circulating miRNA profiles of NFA (n = 4), CPA (n = 5), and PBMAH (n = 5), with respect to tumor type and hormonal activity.

miRNA profiling was conducted on plasma blood samples from 14 patients, and 2652 miRNAs were detected. Unsupervised hierarchical clustering analysis (**Figure 1A**) and principal component analysis (**Figure 1B**) showed that samples of NFA and CPA, both of which were diagnosed as adenomas, clustered into a single group (“adenoma”), in contrast to the more distinct profiles of PBMAH samples. miRNA signatures of PBMAH were classified into two subtypes: subtype 1: including two samples (of patients No. 11 and 14) and subtype 2: including three samples (of patients No. 10, 12, and 13).

The “adenoma” group showed a tendency to discriminate between NFA and CPA. Only one CPA sample (No. 5) was included in the cluster consisting of four NFA samples (**Figure 1A**). Comparison of miRNA profiles based on tumor phenotypes by including combinations such as “adenoma (NFA and CPA) vs PBMAH” and “autonomous cortisol secretion (CPA and PBMAH)” were also performed, and the number of differentially expressed (|fold change| >2.0 and adjusted p <0.05) miRNAs was estimated (**Table 2**). The maximum differentially expressed miRNAs (243) were obtained on comparison of NFA and CPA samples. “Adenoma (NFA and CPA)” versus “PBMAH” analysis identified 135 miRNAs while, “autonomous cortisol secretion (CPA and PBMAH)” versus “NFA” identified 44 differentially expressed miRNAs. As morphological differences such as hyperplasia or adenoma were more discriminative factors than autonomous cortisol secretion ability, we treated NFA and CPA samples under a single “adenoma” group during further analysis.

**Table 2:** Number of differentially expressed miRNAs when comparing tumor phenotypes.

Comparison between tumor phenotypes			Number of differentially expressed miRNAs
CPA	vs	NFA	243
Adenoma (NFA and CPA)	vs	PBMAH	135
NFA	vs	PBMAH	99
CPA	vs	PBMAH	54
Autonomous cortisol secretion (CPA and PBMAH)	vs	NFA	44

The number of differentially expressed (|fold change| >2.0, adjusted p <0.05) miRNAs was calculated for each combination of tumor phenotypes.

NFA, nonfunctioning adenoma; CPA, cortisol-producing adenoma; PBMAH, primary bilateral macronodular adrenal hyperplasia.

Additionally, no differentially expressed miRNAs (|fold change| >2.0 and adjusted *p <*0.05) were discovered in any of the samples during comparison based on clinical features such as sex (male versus female), age (younger than median versus older than median), and body mass index (> 25 kg/m^2^ versus within 18.5 to 25 kg/m^2^) revealed.

### Comparison of miRNA expression between adenoma and PBMAH

**Figure 2:**
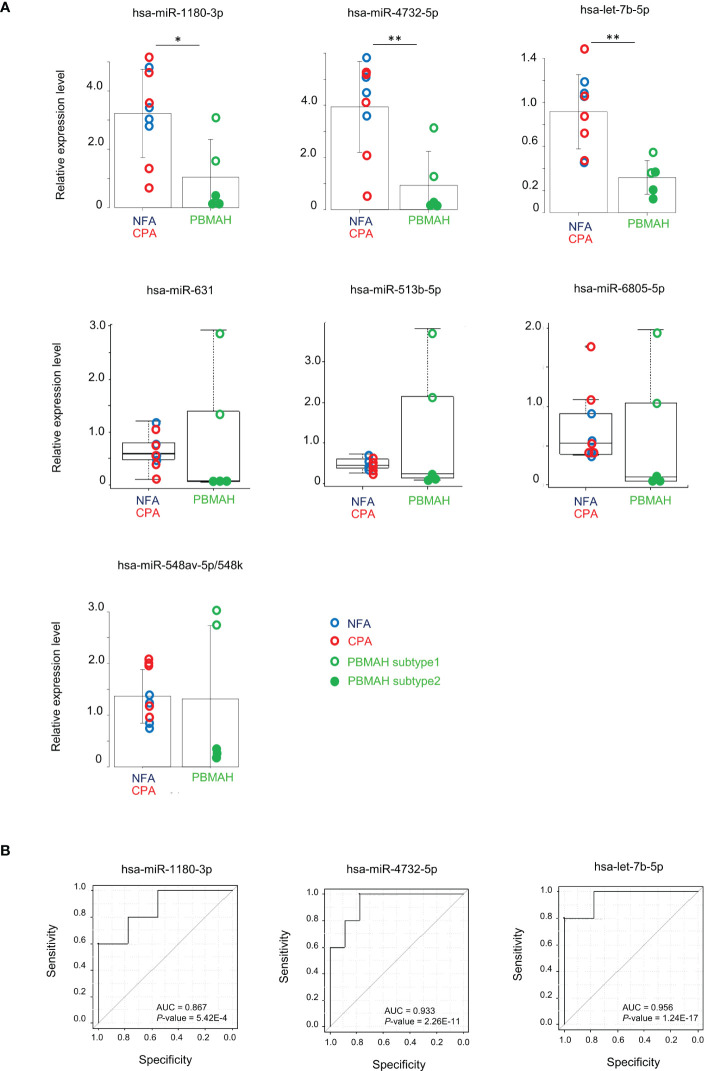
Evaluation of miRNAs expression between adenoma (NFA and CPA) and PBMAH. **(A)** RT-qPCR analysis of hsa-miR-1180-3p, hsa-miR4732-5p, hsa-let-7b-5p, hsa-miR-631, hsa-miR513b-5p, hsa-miR6805-5p, and hsa-miR-548av-5p/548k. Data were normalized to hsa-miR16-5p and are shown with regard to the classification of adenomas including NFA (blue circle, n = 4), CPA (red circle, n = 5), and PBMAH (green circle, n = 5). Patients with PBMAH were divided into two subtypes based on hierarchical clusters. Subtype 1 (green open circles) includes sample numbers 11 and 14, and subtype 2 (green closed circles) includes sample numbers 10, 12, and 13. Student’s *t*-test or Mann-Whitney U test was used for the classification of adenoma versus PBMAH and bar plots with mean ± SD or box plots with the upper and lower quartiles and the median were shown for each analysis, respectively. *P <0.05, **P <0.01. **(B)** ROC curves for the prediction of PBMAH by hsa-miR-1180-3p, hsa-miR4732-5p, and hsa-let-7b-5p. Area under the curve (AUC) and *P-*values are also shown.

Clustering analysis and differentially expression results between miRNAs of tumor phenotypes suggested that circulating miRNA expression could discriminate PBMAH from adenoma. 29 discriminative miRNAs were detected with high average reads (>10) (**Table 3**). Expression analysis of miRNAs in plasma samples from the 14 patients was performed using RT-qPCR. Data are shown with respect to each tumor type (**Figure 2A**). The comparison between “Adenoma (NFA and CPA) and PBMAH” was emphasized and significantly lower expression of hsa-miR-1180-3p and hsa-miR-4732-5p in PBMAH (*P* = 0.019 and 0.006, respectively, **Figure 2A**) was observed. Additionally, we evaluated the expression of hsa-let-7b-5p using RT-qPCR because hsa-let-7b-5p was abundant in the plasma samples (average reads > 230,000) and its relevance to primary pigmented nodular adrenocortical disease (PPNAD), a bilateral adrenal disease with hypercortisolemia has been reported (18). Hsa-let-7b-5p, although not detected as a discriminative miRNA during miRNA profiling (1.92-times downregulated in PBMAH compared to adenoma, adjusted *P* = 0.059), was found to be significantly downregulated in PBMAH (*P* = 0.003, **Figure 2A**) during qRT-PCR. On the other hand, among the miRNAs showing significant differential expression between adenoma and PBMAH in miRNA profiles (**Table 3**), the expressions of hsa-miR-631, hsa-miR-513b-5p, hsa-miR-6805-5p, and hsa-miR-548av-5p/548k were not significantly altered during validation by qRT-PCR (*P* = 0.606, 0.606, 0.438, and 0.926, respectively; **Figure 2A**). The comparison of miRNA expressions between CPA and PBMAH was also performed to focus only on tumors with hormonal activity. The expressions of hsa-miR-1180-3p and hsa-miR4732-5p showed a trend toward a decrease in PBMAH (*P* = 0.093 and 0.061, respectively, **Supplemental Figure 1**), and those of hsa-let-7b-5p showed a significant decrease in PBMAH in spite of small sample size (*P* = 0.013, **Supplemental Figure 1**).

**Table 3:** miRNAs with significantly different expression (adjusted p < 0.05) between PBMAH and adenoma.

miRNAs	Fold change	*P*-value	adjusted *P*-value	PBMAH/adenoma
hsa-miR-4743-3p	0.08	3.79E-07	1.76.E-04	down
hsa-miR-548h-5p	7.04	1.30.E-06	3.66.E-04	up
hsa-miR-8082	0.10	3.01E-06	0.001	down
hsa-miR-7160-3p	0.08	5.24E-06	0.001	down
hsa-miR-593-3p	0.10	1.25E-05	0.001	down
hsa-miR-4711-5p	0.12	4.58E-05	0.003	down
hsa-miR-1180-3p	0.18	6.90E-05	0.004	down
hsa-miR-4732-5p	0.26	1.31E-04	0.006	down
hsa-miR-6753-5p	0.12	1.35E-04	0.006	down
hsa-miR-651-5p	5.08	1.72.E-04	0.007	up
hsa-miR-4799-5p	10.96	2.14.E-04	0.008	up
hsa-miR-631	0.10	3.22E-04	0.012	down
hsa-miR-7114-3p	0.13	3.65E-04	0.013	down
hsa-miR-4485-5p	0.13	3.71E-04	0.013	down
hsa-miR-4476	0.12	5.56E-04	0.018	down
hsa-miR-652-5p	9.12	6.11.E-04	0.019	up
hsa-miR-513b-5p	0.10	7.09E-04	0.020	down
hsa-miR-301a-5p	5.40	0.001	0.027	up
hsa-miR-6805-5p	0.20	0.001	0.027	down
hsa-miR-219a-5p	8.62	0.001	0.028	up
hsa-miR-23c	4.28	0.002	0.036	up
hsa-let-7i-3p	5.92	0.002	0.039	up
hsa-miR-539-3p	4.63	0.002	0.039	up
hsa-miR-548av-5p/548k	4.95	0.002	0.039	up
hsa-miR-32-3p	10.87	0.002	0.040	up
hsa-miR-5580-3p	0.33	0.002	0.041	down
hsa-miR-4508	0.32	0.002	0.042	down
hsa-miR-513b-3p	14.30	0.002	0.043	up
hsa-miR-7159-5p	0.29	0.002	0.045	down

qRT-PCR data for hsa-miR-1180-3p, hsa-miR-4732-5p, and hsa-let-7b-5p predicted the diagnosis of PBMAH with high accuracy (area under the curve (AUC) = 0.867, 0.933, and 0.956; *P* = 5.42E-4, 2.26E-11, and 1.24E-17, respectively; **Figure 2B**).

### Comparison of PBMAH subtypes

**Figure 3:**
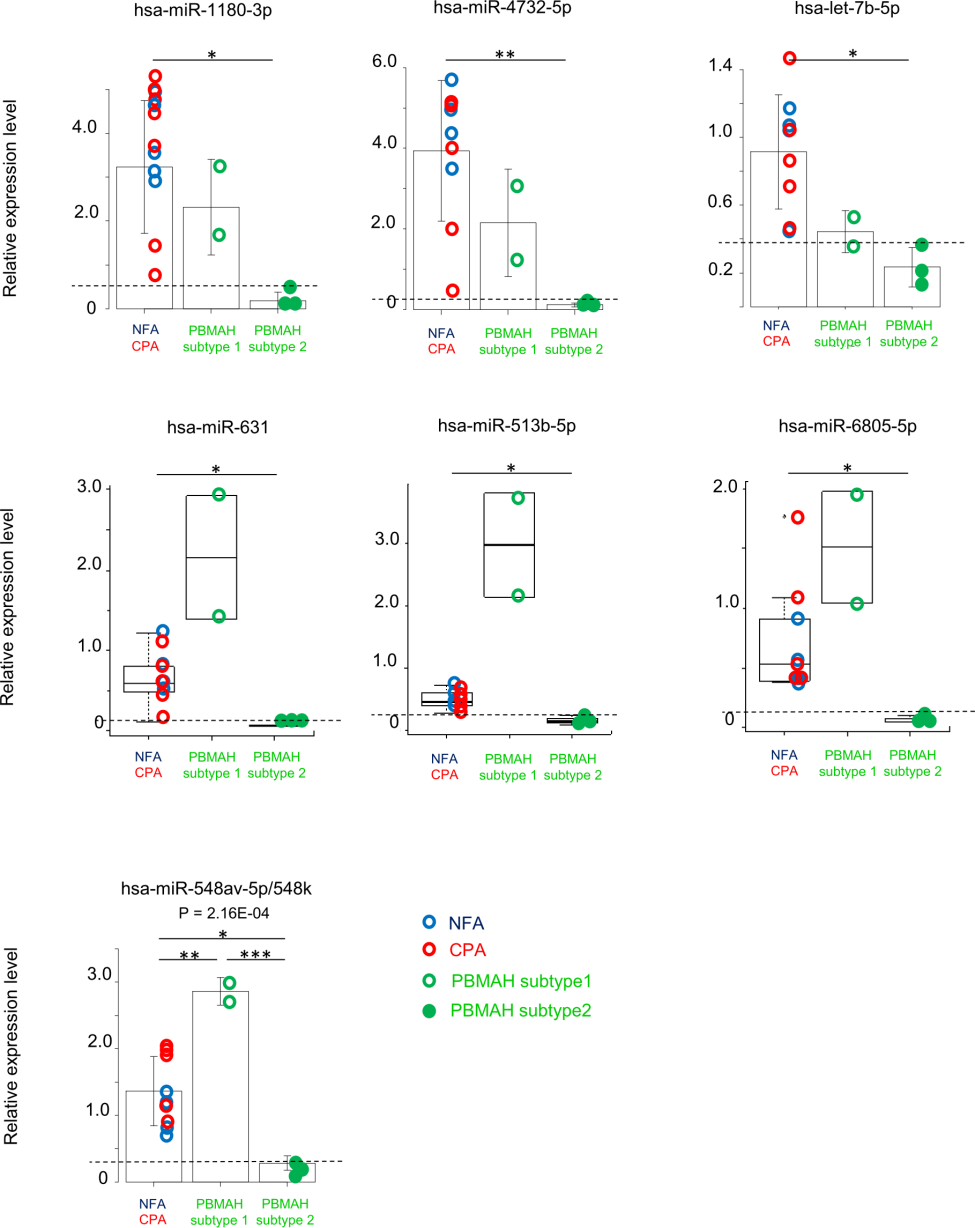
Evaluation of miRNAs expression among the group “adenoma”, “PBMAH subtype 1” and “PBMAH subtype 2”. RT-qPCR analysis of hsa-miR-1180-3p, hsa-miR4732-5p, hsa-let-7b-5p, hsa-miR-631, hsa-miR513b-5p, hsa-miR6805-5p, and hsa-miR-548av-5p/548k. Data were normalized to hsa-miR16-5p and shown with regard to the classification of “adenoma” (NFA: blue circle, n = 4, and CPA: red circle, n = 5), “PBMAH subtype 1” (green open circle, n = 2)” and “PBMAH subtype 2” (green closed circle, n = 3). One-way ANOVA followed by Tukey’s post Hoc test or Kruskal–Wallis test followed by Dunn test were used and bar plots with mean ± SD or box plots with the upper and lower quartiles and the median were shown for each analysis, respectively. *P <0.05, **P <0.01, ***P <0.001. Broken lines indicate the maximum value of PBMAH subtype 2 for reference.

Clustering analysis of the circulating miRNA profiles revealed two subtypes of PBMAH. Subtype 1 profile was more similar to that of adenoma (NFA and CPA) than that of subtype 2 (**Figures 1A, B**). Hence, a comparison among “adenoma”, “PBMAH subtype 1” and “PBMAH subtype 2” was performed. Discriminative miRNAs between adenomas and PBMAH (**Figure 2**) were estimated again with this classification. Expressions of hsa-miR-1180-3p, hsa-miR-4732-5p, hsa-let-7b-5p, hsa-miR-631, hsa-miR-513b-5p, hsa-miR-6805-5p, and hsa-miR-548av-5p/548k were found to be significantly lower in PBMAH subtype 2 than those in “adenoma” (*P* = 0.014, 0.009, 0.013, 0.027, 0.027, 0.027, and 0.010, respectively; **Figure 3**). Expression of those miRNAs were also found to be lower in PBMAH subtype 2 than those in “PBMAH subtype 1” although statistically significance was only observed in the expression levels of hsa-miR-548av-5p/548k (*P* = 1.53E-04) due to small sample size. The comparison of miRNA expressions among CPA, PBMAH subtype 1 and PBMAH subtype 2 was also performed to focus only on tumors with hormonal activity. In comparison to CPA, the expressions of hsa-miR-1180-3p, hsa-miR4732-5p, hsa-miR-631 and hsa-miR-513b-5p showed a trend toward a decrease in PBMAH (*P* = 0.088, 0.075, 0.110 and 0.110, respectively, **Supplemental Figure 2**), and those of hsa-let-7b-5p, hsa-miR-6805-5p and hsa-miR-548av-5p/548k showed a significant decrease in PBMAH subtype 2 in spite of small sample size (*P* = 0.042, 0.043 and 0.007, respectively, **Supplemental Figure 2**).

**Figure 4:**
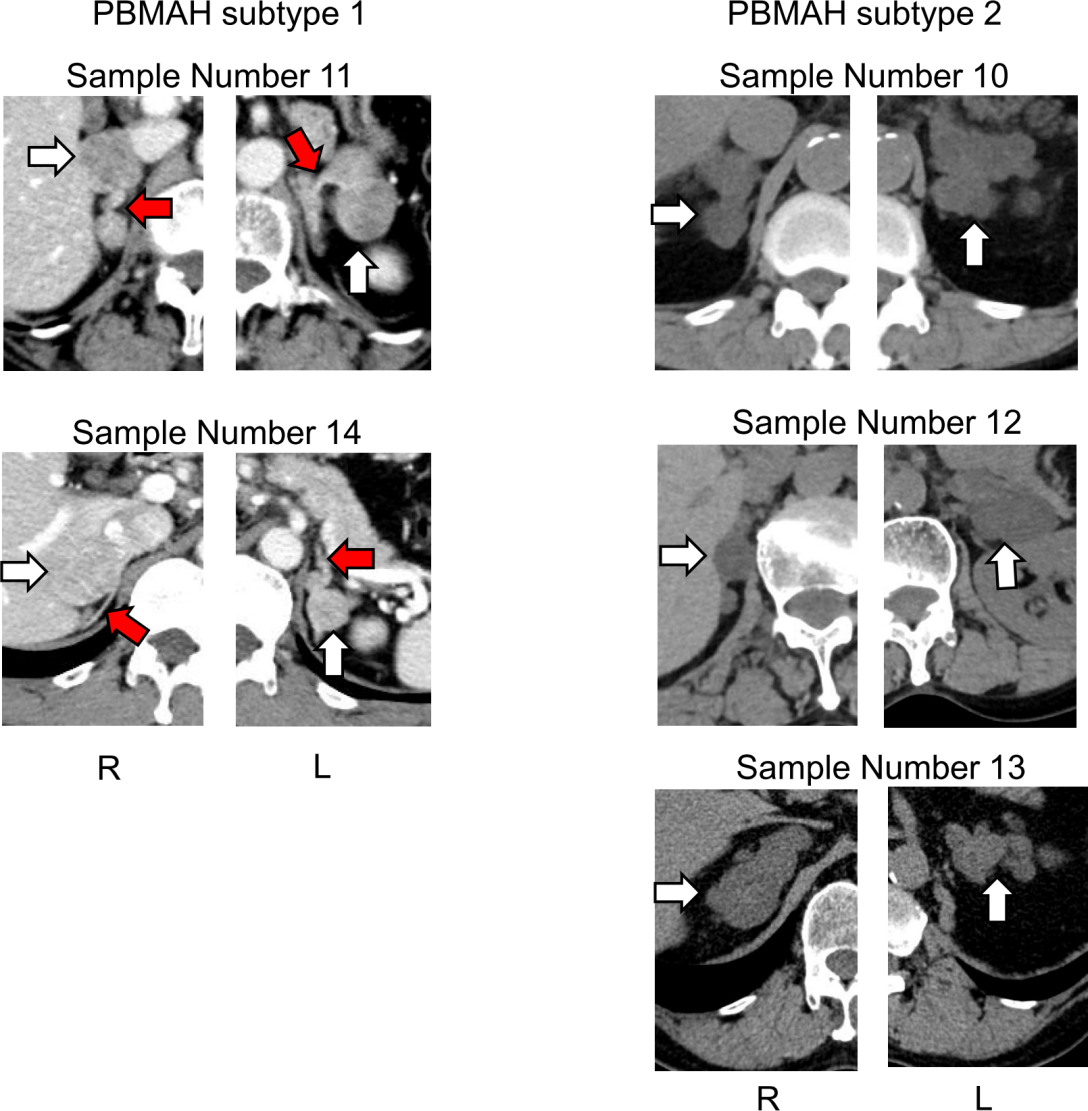
Images of adrenal glands of patients with PBMAH. White arrows indicate adrenal lesions. Red arrows show suspected adrenal tissue lesions without hyperplasia in patients with PBMAH subtype 1.

Although clinical characteristics such as age, sex, BMI, hormonal activity, tumor size and cortisol value after LDDSAT between PBMAH subtype 1 and 2 was examined and found all parameters overlapped between subtypes (**Supplemental Figure 3**), the computed tomography (CT) images suggested morphological differences between PBMAH subtypes 1 and 2. The adrenal mass of subtype 1 contained both multiple adenomas and adjacent adrenal tissues without hyperplasia; in contrast, those of subtype 2 was constituted entirely by adrenal nodular hyperplasia (**Figure 4**).

### Pathway analysis of discriminative miRNAs

Target gene and pathway analyses were performed to obtain insights into miRNA functions. The PBMAH-specific circulating miRNAs hsa-miR-1180-3p, hsa-miR-4732-5p, and hsa-let-7b-5p were examined individually and as combinations in the miR+pathway server. Pathway analysis of hsa-miR-4732-5p extracted “ErbB signaling pathway,” “Melanoma,” and “Chronic myeloid leukemia” (*P* = 0.015, 0.042 and 0.047, respectively, **Table 4A**). These pathways comprised *MAP2K7, ERBB3, MAP2K2, CDKN1A*, and *TGFBR2* as target genes. The combination of hsa-miR-4732-5p and hsa-miR-1180-3p as an input for pathway analysis provided “thyroid cancer” as an altered pathway (*P* = 0.037, **Table 4A**), including *MAP2K2*, *CDKN1A*, and *TRP* as target genes. Pathways of discriminative miRNAs for PBMAH subtype 2, focusing on hsa-miR-631, hsa-miR-513b-5p, hsa-miR-6805-5p, and hsa-miR-548av-5p/548k, were also analyzed. When using hsa-miR-4732-5p, pathway analysis extracted “Antifolate resistance” (*P* = 0.008, **Table 4B**) as an altered pathway that included *SHMT1*, *SHMT2*, and *ABCC5* as target genes. “Small cell lung cancer” was also extracted (*P* = 0.040, **Table 4B**) and included *RXRB, E2F1, CDKN1A*, and *TRAF3*. The combination of hsa-miR-6805-5p and hsa-miR-513b-5p for pathway analysis also provided “Antifolate resistance” (*P* = 0.016, **Table 4B**) as the resultant pathway with *SHMT1*, *SHMT2*, *ABCC5*, and *IKBKG* as the target genes.

**Table 4:** Pathway analysis of discriminative miRNAs for PBMAH and PBMAH subtype 2.

A			
Pathway analysis results for PBMAH-specific miRNAs: hsa-miR-1180-3p, hsa-miR-4732-5p, and hsa-let-7b-5p.			
miRNA	Pathway	*P*-value	Target Genes
hsa-miR-4732-5p	ErbB signaling pathway	*P*-value	Target Genes
	Melanoma	0.042	MAP2K2, ERBB3, CDKN1A
	Chronic myeloid leukemia	0.047	MAP2K2, ERBB3, CDKN1A, TGFBR2
hsa-miR-4732-5p	Thyroid cancer	0.037	MAP2K2, CDKN1A
hsa-miR-1180-3p			TPR
B			
Pathway analysis results for PBMAH subtype2-specific miRNAs: hsa-miR-631, hsa-miR-513b-5p, hsa-miR-6805-5p, and hsa-miR-548av-5p/548k.			
miRNA	Pathway	*P*-value	Target Genes
hsa-miR-6805-5p	Antifolate resistance	0.008	SHMT1, SHMT2, ABCC5
	Small cell lung cancer	0.040	RXRB, E2F1, CDKN1A, TRAF3
hsa-miR-6805-5p	Antifolate resistance	0.016	SHMT1, SHMT2, ABCC5
hsa-miR-513b-5p			IKBKG

## Discussion

This is the first study to compare the circulating miRNA profiles of PBMAH with that of adenomas with and without cortisol excess. The analysis enabled us to elucidate the differences between the circulating miRNA profiles of PBMAH and adenomas. miRNAs, such as hsa-miR-1180-3p, hsa-miR-4732-5p, and hsa-let-7b-5p, were downregulated in PBMAH. In addition, we revealed heterogeneity in PBMAH samples based on circulating miRNAs and accordingly identified profiling-based subtypes. In addition to the previously mentioned hsa-miR-1180-3p, hsa-miR-4732-5p, and hsa-let-7b-5p miRNAs, the subtype 2-specific miRNAs, hsa-miR-631, hsa-miR-513b-5p, hsa-miR-6805-5p, and hsa-miR-548av-5p/548k were downregulated, hence, we hypothesized that the subtypes observed based on circulating miRNAs might be in accordance with the morphological heterogeneity of PBMAH (2).

A previous study has demonstrated PBMAH-specific miRNAs in adrenal tissues (17). A comparison between tissues with massive macronodular adrenocortical disease, which corresponded to PBMAH, and normal adrenal glands extracted 37 differentially expressed miRNAs out of the total detected 365 miRNAs (17). Another similar study that explored miRNA expression in PBMAH tissues compared to normal adrenal glands identified 16 and 8 differentially expressed miRNAs in familial and sporadic phenotypes, respectively (19). In both these studies, there were no common miRNAs that showed PBMAH-specific expression, and additionally the miRNA hsa-miRNA-130a even showed opposing results in both. With regards to the statistical tests, previous studies adopted only fold-change or non-adjusted *P*-values to extract specific miRNAs. It seemed difficult to find an apparent discrimination between the miRNA profiles of PBMAH tissues and normal adrenal glands from tissue samples. In contrast, our analysis focused on circulating miRNA profiles of patients with NFA, CPA, and PBMAH and successfully demonstrated clear distinction between PBMAH and adenoma (NFA and CPA) phenotypes by clustering analysis. Our analysis showed that NFA and CPA were distinct from each other with 243 differentially expressed circulating miRNAs. However, the PBMAH group showed a higher degree of separation from both NFA and CPA during hierarchical clustering and principal component analysis. It is noteworthy that comparison focused on “autonomous cortisol secretion (CPA and PBMAH) versus NFA” provided only fewer differentially expressed miRNAs (one-third of what was obtained from “PBMAH versus adenoma” analysis). Thus, during circulating miRNA profiling of benign adrenal neoplasms including PBMAH, cortisol secretion ability seemed to be a less important factor than morphological differences, such as hyperplasia and adenoma.

Based on the previous article regarding blood miRNA signatures in CPA (16), hsa-miR-22-3p, hsa-miR-27a-3p and hsa-miR-320b were significantly upregulated in samples of CPA compared to those of NFA and our data showed that hsa-miR-22-3p showed a tendency to be upregulated in samples of CPA compared to those of NFA (fold change = 1.92, P-value = 0.033, adjusted P-value = 0.361). Hsa-miR-27a-3p and hsa-miR-320b showed upregulation in CPA without statistically significance (fold change = 1.99 and 1.76, P-value = 0.099 and 0.206, adjusted P-value = 0.511 and 0.607, respectively). Accurate overlap was not observed; however, all miRNAs were consistently “upregulated” in CPA in both studies. It was speculated that the difference of miRNA extraction method, in which miRNAs were extracted from extracellular vesicle in the previous article, would affect the results

Some genetic information on PBMAH, such as the nature of *ARMC5* mutation, is known. Germline heterozygous mutations are present in the *ARMC5* gene of 10% to 55% of patients with PBMAH, and the second somatic inactivating mutation in the *ARMC5* gene in adrenal hyperplasia tissue suggests that this gene acts as a tumor suppressor gene (4, 29, 30). Previous studies on non-small cell lung carcinoma demonstrated that circulating miRNA expression signature was associated with the existence of tumor-specific mutation status, such as the *EGFR* gene mutation (31, 32) It can be hence presumed that genetic alteration in PBMAH would affect tissue miRNA expression and systemic response due to adrenal hyperplasia formation and induce unique circulating miRNA profiles.

Our analysis revealed that the three circulating miRNAs could discriminate PBMAH from adenomas. The miRNA pathway analysis revealed that hsa-miR-4732-5p has putative downstream targets involved in the melanoma, chronic myeloid leukemia, and ErbB signaling pathways. Thus, our results suggest that pathways related to malignant tumors were affected in Patients with PBMAH, although PBMAH is considered a pathologically benign disease without a propensity for invasion and metastasis. Interestingly, consistent with a previous study that investigated the miRNA profile of PBMAH tissues and predicted the involvement of MAPK and TGF-β signaling pathways (6), putative targets of hsa-miR-4732-5p comprised genes related to the MAPK signaling (*MAP2K2* and *MAP2K7*) and TGF-β signaling (*TGFBR2*) pathways. Further laboratory investigation is expected to dissect the pathophysiological roles of the MAPK and TGF-β signaling pathways in PBMAH because of their association with cell development and cell cycle regulation (33), and involvement of the TGF-β signaling pathway in steroidogenesis of the human adrenal cell line (34). Low abundance was observed for hsa-miR-1180-3p and hsa-let-7b-5p. Hsa-miR-1180-3p was downregulated in the blood samples of non-functioning pituitary adenomas compared to that in healthy subjects (35). Although the functional role of hsa-miR-1180-3p is unknown, alterations in the blood samples of endocrine neoplasms including PBMAH and pituitary adenoma, suggests its involvement in the development of specific endocrine diseases. Hsa-let-7b-5p was reported to be a core miRNA in the regulation of candidate genes involved in glioma development and was suggested to inhibit the migration, invasion, and cell cycle of glioma cells (36). Downregulation of hsa-let-7b-5p may be related to adrenal hyperplasia.

One of the important findings in our analysis was the heterogeneity of the circulating miRNA profiles of PBMAHs. Based on clustering analysis, we documented two subtypes in PBMAH samples, and the profiles of subtype 1 were more similar to those of the adenoma phenotype. The similarities between the subtype 1 and adenoma groups were also validated using qPCR data. Although clinical features such as cortisol excess did not explain the subtyping, information from CT images was speculative. CT images of PBMAH subtype 1 consisted of multiple adenomas with possibly atrophic adjacent adrenal glands, whereas those of PBMAH subtype 2 showed diffuse hyperplasia. It should be noted that the characteristics of subtype 1 with multiple adenomas may contribute to the similarity of its miRNA profiles to those of the adenoma group than those of subtype 2. Our subtyping, based on comprehensive miRNA profiling, seemed to correspond to known pathological subgroups: multiple adenomas with inter-nodular atrophic cortical tissue and diffuse multinodular hyperplasia (9, 10). As pathological subgroups should be evaluated at the microscopic histological level, our proposal remains a matter of speculation. Nevertheless, it can be presumed that circulating miRNA profile reflects the heterogeneity of the molecular pathophysiology of PBMAH. In addition, pathway analysis of PBMAH subtype2-specific miRNA extracted “Antifolate resistance” including *SHMT1* and *SHMT2* genes as target genes of hsa-miR-6805-5p. *SHMT1* and *SHMT2* both encode serine hydroxymethyltransferase, which is a pyridoxal phosphate-dependent enzyme responsible for regulating the serine/glycine one−carbon metabolic network. SHMT catalyzes the conversion of serine to glycine, simultaneously hydrolyzing 5,10-tetrahydrofolate (THF) into methylene-THF and accelerating cell proliferation (37, 38). The ectopic expression of *SHMT* genes has been reported in various human cancers as an oncogenic driver. It is conceivable that unique cell proliferation mechanisms involving *SHMT1* and *SHMT2* gene expression through the downregulation of hsa-miR-6805-5p are relevant to the pathophysiology of PBMAH subtype 2.

In the future, the identification of different subtypes by circulating miRNA profiling has the potential to support the choice for adrenal surgical methods. Bilateral adrenalectomy or removal of larger adrenal glands is often performed. It is also known to use image techniques such as scintigraphy to decide which side of adrenal glands to be removed (39), however, the information is only about the size or hormonal activity of adrenal disease and not able to suggest the partial adrenalectomy. The current evidence regarding the use of partial adrenalectomy is limited (40). If circulating miRNA expression can predict the subtypes of PBMAH in addition to imaging information, partial adrenalectomy could be adopted more often in cases of multiple adenoma types, which can’t be distinguished from hyperplasia types using scintigraphy.

Our study has some limitations. First, our cohort included only five patients with PBMAH. We should prepare additional samples from PBMAH patients to confirm reproducibility, after that, a study including a larger number of patients would be planned to validate our data in the future, although our cohort was comparable to that in a previous study of PBMAH and provided unique circulating miRNA profiles in relation to adrenal disease phenotype. Second, pathological specimens of PBMAH samples were not obtained and analyzed in the study as no patients with PBMAH were surgically treated. The relationship between the miRNA profiles from adrenal tissues and those of circulating miRNAs should be examined to evaluate their pathophysiological significance. In addition, detailed pathological diagnoses, such as multiple adenomas or hyperplasia, could provide more insights into our hypothesis regarding subtyping based on circulating miRNA profiles. Finally, the genomic ARMC5 mutation status was not asserted in our cohort. It is possible to say that genomic information would be useful for understanding the molecular basis of the heterogeneity of PBMAH, as observed during circulating miRNA profiling.

## Conclusion

We identified signatures of circulating miRNA specific to patients with PBMAH. We hypothesized that miRNA profiling subtypes are associated with the pathophysiological heterogeneity of PBMAH.

## Data availability statement

The data used in the present publication have been deposited in the National Center for Biotechnology Information Gene Expression Omnibus and are accessiblethrough Gene Expression Omnibus (series accession no. GSE220070 for gene expression array).

## Ethics statement

The studies involving human participants were reviewed and approved by Tokyo Medical and Dental University, Kyoto University National Hospital Organization, and Kyoto Medical Center. The patients/participants provided their written informed consent to participate in this study. Written informed consent was obtained from the individual(s) for the publication of any potentially identifiable images or data included in this article.

## Author contributions

MN and MM designed this study. KH, MM, MH, YN, ATak, KS, MH, KT, CK, KI, MT, MY, YF, and TY recruited patients and collected clinical data. KH, MM, ATan, MN, and TT performed miRNA and statistical analyses of the results. KH, MM, and MN drafted the manuscript. All authors contributed to writing the manuscript and approved the version to be published.
