# Supplementary material for: Heterogeneous circulating miRNA profiles of PBMAH
Source: Front Endocrinol (Lausanne). 2022 Dec 13;13:1073328. doi: 10.3389/fendo.2022.1073328 (PMC9792611; doi:10.3389/fendo.2022.1073328)
Supplement: Supplementary file 1 [file Presentation_1.pptx]

## Slide 1
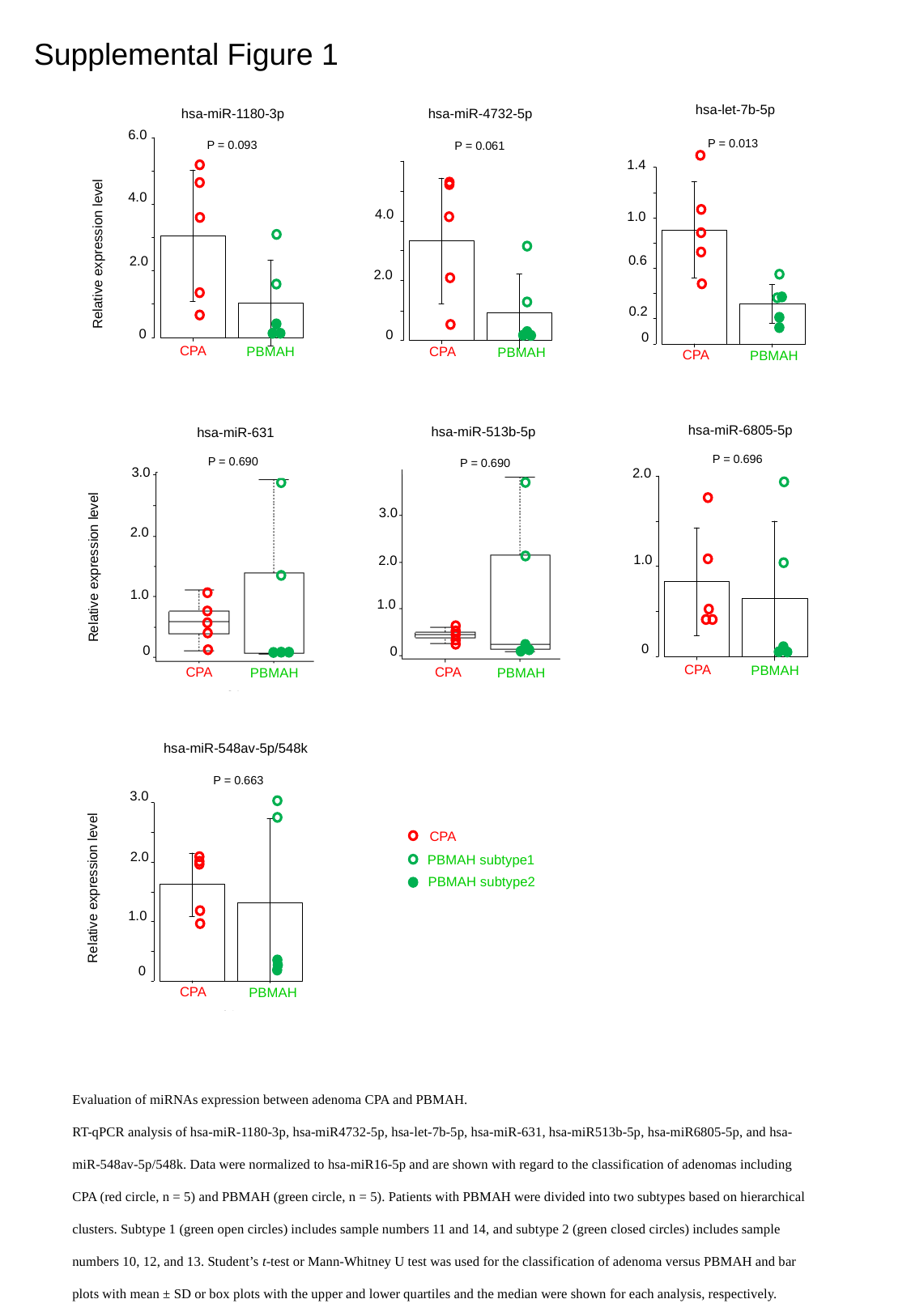

Supplemental Figure 1
hsa-let-7b-5p
hsa-miR-4732-5p
hsa-miR-1180-3p
6.0
P = 0.013
P = 0.093
P = 0.061
1.4
4.0
4.0
1.0
Relative expression level
0.6
2.0
2.0
0.2
0
0
0
CPA
CPA
PBMAH
PBMAH
CPA
PBMAH
hsa-miR-6805-5p
hsa-miR-513b-5p
hsa-miR-631
P = 0.696
P = 0.690
P = 0.690
3.0
2.0
3.0
2.0
1.0
2.0
Relative expression level
1.0
1.0
0
0
0
CPA
PBMAH
CPA
CPA
PBMAH
PBMAH
hsa-miR-548av-5p/548k
P = 0.663
3.0
CPA
2.0
PBMAH subtype1
PBMAH subtype2
Relative expression level
1.0
0
CPA
PBMAH
Evaluation of miRNAs expression between adenoma CPA and PBMAH.
RT-qPCR analysis of hsa-miR-1180-3p, hsa-miR4732-5p, hsa-let-7b-5p, hsa-miR-631, hsa-miR513b-5p, hsa-miR6805-5p, and hsa-miR-548av-5p/548k. Data were normalized to hsa-miR16-5p and are shown with regard to the classification of adenomas including CPA (red circle, n = 5) and PBMAH (green circle, n = 5). Patients with PBMAH were divided into two subtypes based on hierarchical clusters. Subtype 1 (green open circles) includes sample numbers 11 and 14, and subtype 2 (green closed circles) includes sample numbers 10, 12, and 13. Student’s t-test or Mann-Whitney U test was used for the classification of adenoma versus PBMAH and bar plots with mean ± SD or box plots with the upper and lower quartiles and the median were shown for each analysis, respectively.

## Slide 2
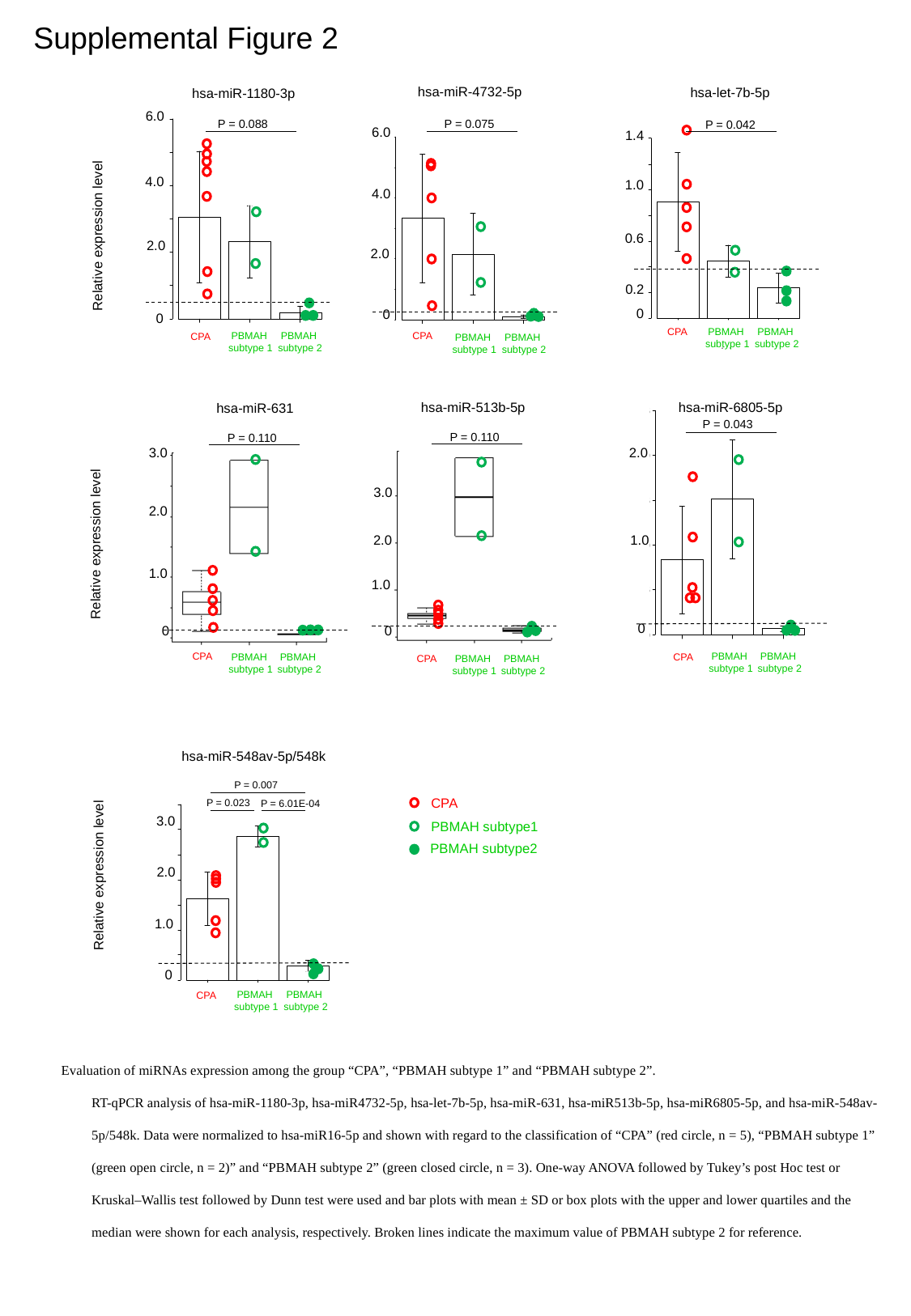

Supplemental Figure 2
hsa-miR-4732-5p
hsa-let-7b-5p
hsa-miR-1180-3p
6.0
P = 0.075
P = 0.088
P = 0.042
6.0
1.4
4.0
1.0
4.0
Relative expression level
0.6
2.0
2.0
0.2
0
0
0
PBMAH
subtype 2
PBMAH
subtype 1
CPA
PBMAH
subtype 2
PBMAH
subtype 1
CPA
CPA
PBMAH
subtype 2
PBMAH
subtype 1
hsa-miR-513b-5p
hsa-miR-6805-5p
hsa-miR-631
P = 0.043
P = 0.110
P = 0.110
3.0
2.0
3.0
2.0
1.0
2.0
Relative expression level
1.0
1.0
0
0
0
PBMAH
subtype 2
PBMAH
subtype 1
CPA
CPA
PBMAH
subtype 2
PBMAH
subtype 1
CPA
PBMAH
subtype 2
PBMAH
subtype 1
hsa-miR-548av-5p/548k
P = 0.007
CPA
P = 0.023
P = 6.01E-04
3.0
PBMAH subtype1
PBMAH subtype2
2.0
Relative expression level
1.0
0
PBMAH
subtype 2
PBMAH
subtype 1
CPA
Evaluation of miRNAs expression among the group “CPA”, “PBMAH subtype 1” and “PBMAH subtype 2”.
RT-qPCR analysis of hsa-miR-1180-3p, hsa-miR4732-5p, hsa-let-7b-5p, hsa-miR-631, hsa-miR513b-5p, hsa-miR6805-5p, and hsa-miR-548av-5p/548k. Data were normalized to hsa-miR16-5p and shown with regard to the classification of “CPA” (red circle, n = 5), “PBMAH subtype 1” (green open circle, n = 2)” and “PBMAH subtype 2” (green closed circle, n = 3). One-way ANOVA followed by Tukey’s post Hoc test or Kruskal–Wallis test followed by Dunn test were used and bar plots with mean ± SD or box plots with the upper and lower quartiles and the median were shown for each analysis, respectively. Broken lines indicate the maximum value of PBMAH subtype 2 for reference.

## Slide 3
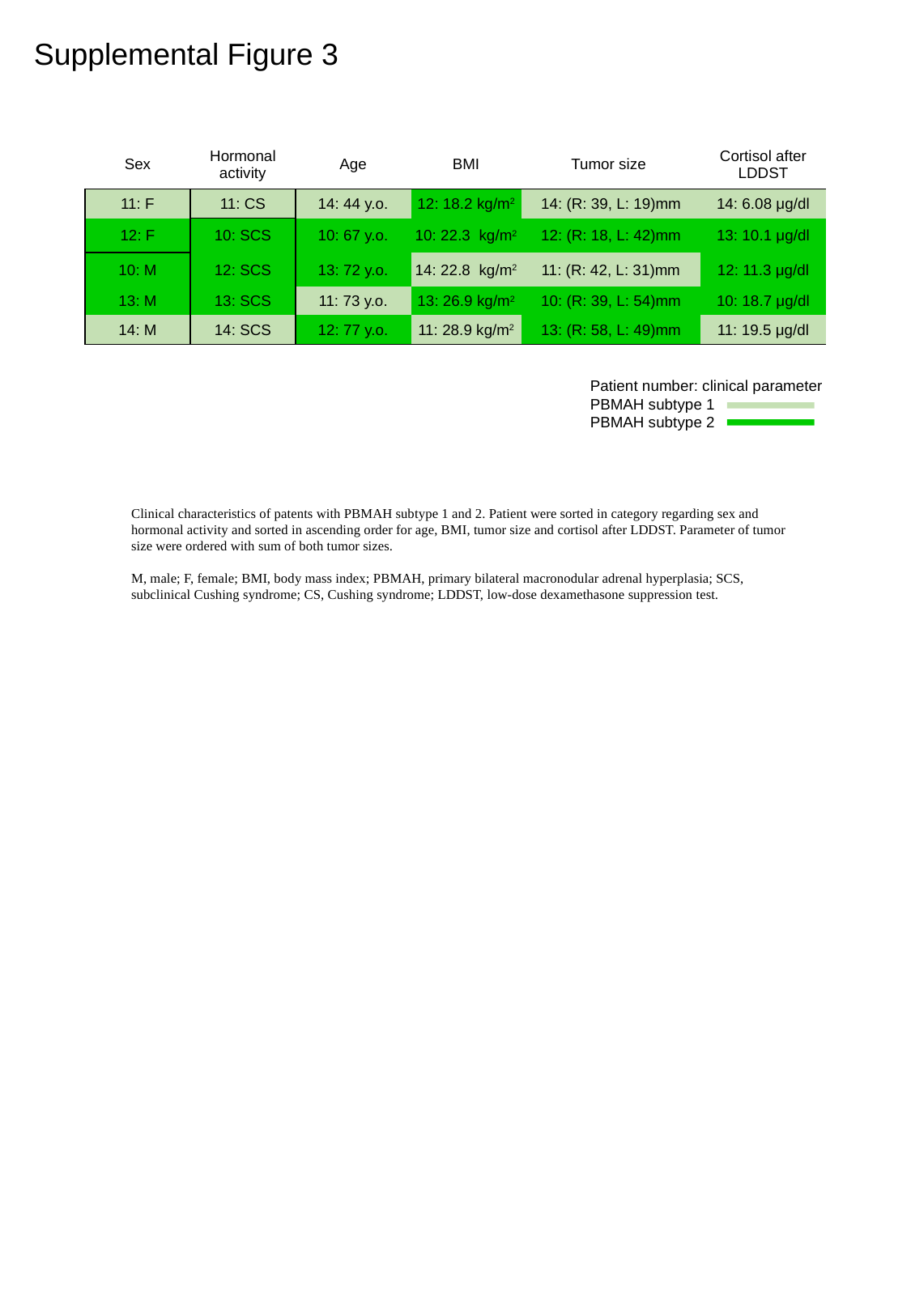

Supplemental Figure 3
| Sex | Hormonal activity | Age | BMI | Tumor size | Cortisol after LDDST |
| --- | --- | --- | --- | --- | --- |
| 11: F | 11: CS | 14: 44 y.o. | 12: 18.2 kg/m2 | 14: (R: 39, L: 19)mm | 14: 6.08 μg/dl |
| 12: F | 10: SCS | 10: 67 y.o. | 10: 22.3 kg/m2 | 12: (R: 18, L: 42)mm | 13: 10.1 μg/dl |
| 10: M | 12: SCS | 13: 72 y.o. | 14: 22.8 kg/m2 | 11: (R: 42, L: 31)mm | 12: 11.3 μg/dl |
| 13: M | 13: SCS | 11: 73 y.o. | 13: 26.9 kg/m2 | 10: (R: 39, L: 54)mm | 10: 18.7 μg/dl |
| 14: M | 14: SCS | 12: 77 y.o. | 11: 28.9 kg/m2 | 13: (R: 58, L: 49)mm | 11: 19.5 μg/dl |
Patient number: clinical parameter
PBMAH subtype 1
PBMAH subtype 2
Clinical characteristics of patents with PBMAH subtype 1 and 2. Patient were sorted in category regarding sex and hormonal activity and sorted in ascending order for age, BMI, tumor size and cortisol after LDDST. Parameter of tumor size were ordered with sum of both tumor sizes.
M, male; F, female; BMI, body mass index; PBMAH, primary bilateral macronodular adrenal hyperplasia; SCS, subclinical Cushing syndrome; CS, Cushing syndrome; LDDST, low-dose dexamethasone suppression test.
